# Cost-Effectiveness Analysis of Age-Specific N-Terminal Pro-B-Type Natriuretic Peptide Thresholds for Heart Failure Diagnosis in China: Protocol for a Markov Model–Based Study

**DOI:** 10.2196/95071

**Published:** 2026-06-29

**Authors:** Changyuan Liu, Jing Wang, Shuangning Ding, Yongze Li

**Affiliations:** 1 Department of Endocrinology and Metabolism and the Institute of Endocrinology National Health Commission Key Laboratory of Diagnosis and Treatment of Thyroid Diseases First Hospital of China Medical University Shenyang, Liaoning China

**Keywords:** heart failure, N-terminal pro-B-type natriuretic peptide, NT-proBNP, cost-effectiveness analysis, thresholds, Markov model, diagnostic strategy

## Abstract

**Background:**

Heart failure (HF) imposes a substantial clinical and economic burden in China. Although N-terminal pro-B-type natriuretic peptide (NT-proBNP) is widely used in the diagnostic pathway for HF, conventional thresholds are largely derived from Western populations and may not be fully applicable to Chinese adults.

**Objective:**

This study aims to evaluate the cost-effectiveness of an exploratory age-specific NT-proBNP threshold strategy compared with a conventional universal threshold strategy for HF diagnosis in China.

**Methods:**

We developed a hybrid decision-analytic model linking an initial diagnostic decision tree to long-term Markov state-transition pathways. The target population comprised adults aged ≥45 years with symptoms suggestive of HF who underwent further clinical diagnostic evaluation. The China Health and Retirement Longitudinal Study was used to inform the baseline demographic and comorbidity structure. Two diagnostic strategies were compared: (1) an exploratory age-specific NT-proBNP strategy using thresholds of 100 ng/L for individuals aged <65 years and 248 ng/L for those aged ≥65 years and (2) a conventional threshold strategy using 125 ng/L as the comparator cutoff. The analysis was conducted from the perspective of the Chinese health care system. Primary outcomes included direct medical costs, quality-adjusted life-years, and incremental cost-effectiveness ratios. The base-case analysis used a 10-year time horizon, with annual discounting of 5% for both costs and health outcomes. Parameter uncertainty will be assessed using deterministic sensitivity analysis, probabilistic sensitivity analysis with 5000 Monte Carlo simulations, and scenario analyses.

**Results:**

This study was funded in January 2025. Model construction has been completed, and parameter collection, calibration, and validation are ongoing. The final cost-effectiveness analysis is expected to be completed after project-specific Chinese data become available. Planned outputs include total costs, quality-adjusted life-years, incremental cost-effectiveness ratios, diagnostic classification outcomes, and cost-effectiveness acceptability results.

**Conclusions:**

This protocol will assess whether age-specific NT-proBNP thresholds provide economic or clinical advantages over a conventional universal threshold strategy in the Chinese health care setting. The findings are intended to inform future diagnostic policy while explicitly acknowledging uncertainty surrounding the exploratory age-specific thresholds and other structural assumptions.

**International Registered Report Identifier (IRRID):**

PRR1-10.2196/95071

## Introduction

Heart failure (HF) is an increasingly important public health problem in China, driven by rapid population aging and the rising prevalence of cardiovascular risk factors among middle-aged and older adults. In 2023, the number of individuals living with HF in China was estimated at approximately 14.3 million, representing a 208.4% increase since 1990 [1,2]. HF is associated with a substantial clinical and economic burden. Hospitalizations account for a large share of direct medical costs and contribute importantly to health care expenditure and resource use. In addition, the cumulative economic burden attributable to HF from 2025 to 2035 has been estimated at INT $1001.1 billion [3]. The poor prognosis of HF, particularly after diagnosis or hospitalization, including the high risks of death and rehospitalization, highlights the need for more efficient diagnostic and management strategies [4].

N-terminal pro-B-type natriuretic peptide (NT-proBNP), a biomarker of myocardial wall stress, is widely used in the diagnostic evaluation of HF and is closely associated with disease severity and prognosis [5]. The National Academy of Clinical Biochemistry and the European Society of Cardiology recommend NT-proBNP as an important laboratory marker for the diagnosis and management of HF [6,7]. However, the reference intervals and decision thresholds currently applied in China are still largely influenced by manufacturer-derived values and laboratory practices rather than by prospectively validated diagnostic threshold studies conducted in Chinese populations [8,9]. Consequently, commonly used universal cutoffs may not fully reflect ethnic and physiologic differences in NT-proBNP distributions among Chinese adults.

Emerging evidence from Chinese populations indicates substantial age-related variation in NT-proBNP concentrations. Shi et al [10] reported that, among 587 apparently healthy Chinese individuals, the NT-proBNP reference range showed clear age dependence, with an upper limit of <114 ng/L in those aged ≤60 years, which was substantially lower than the manufacturer-recommended threshold. Similarly, a doctoral dissertation [11] reported that NT-proBNP concentrations encompassing at least 90% of apparently healthy Chinese adults corresponded to <100 ng/L for those aged <65 years and <248 ng/L for those aged ≥65 years. These values differ considerably from the commonly used universal cutoff of 125 ng/L. Together, these findings suggest that age-adjusted NT-proBNP thresholds may improve diagnostic precision and help optimize health care resource use in Chinese adults. However, these thresholds have not been directly validated in a dedicated cohort of symptomatic Chinese patients with suspected HF and have not been incorporated into national HF guidelines as standard diagnostic cutoffs [9].

This protocol describes a model-based economic evaluation comparing an exploratory age-specific NT-proBNP threshold strategy with a conventional universal threshold strategy in China. The study is intended to assess whether age-specific thresholds could improve diagnostic classification and health care resource use while explicitly acknowledging uncertainty related to threshold selection and other structural assumptions [12].

## Methods

### Model Overview

We developed a hybrid decision-analytic model to evaluate the cost-effectiveness of alternative NT-proBNP threshold strategies in adults with symptoms suggestive of HF. The model consisted of a short-term diagnostic decision tree followed by long-term Markov state-transition pathways. The decision tree captured initial diagnostic classification, whereas the Markov component represented the downstream clinical and economic consequences of correct diagnosis, delayed diagnosis, unnecessary referral, hospitalization, and death [13].

We used a cohort-based state-transition model linked to an initial diagnostic decision tree rather than a transmission-dynamic model because the diagnostic and clinical trajectories of symptomatic individuals do not depend on interactions between individuals. Although patients transitioned between health states over time, the model was static in the epidemiologic sense because transition probabilities were not influenced by interpersonal contact, transmission processes, or population density [12,14]. The decision tree determined only the initial diagnostic classification; thereafter, patients progressed through the corresponding downstream Markov pathway and did not re-enter the initial diagnostic phase in subsequent cycles [14]. The CHEERS (Consolidated Health Economic Evaluation Reporting Standards) 2022 checklist is provided in Multimedia Appendix 1.

### Target Population and Diagnostic Strategies

The target population comprised adults aged ≥45 years with symptoms suggestive of HF who underwent further clinical diagnostic evaluation. The China Health and Retirement Longitudinal Study was used to inform the baseline demographic and comorbidity structure of Chinese adults aged ≥45 years, but it was not used to directly represent the symptomatic suspected-HF cohort [15]. Specifically, the China Health and Retirement Longitudinal Study provided the baseline distribution of age; sex; and major comorbidities, including hypertension, diabetes, coronary heart disease, and other relevant chronic conditions, among Chinese adults aged ≥45 years. The simulated suspected-HF cohort was then constructed by combining this baseline structure with symptom-based assumptions and calibration targets derived from Chinese HF registries and published literature, including estimates of underlying HF prevalence, hospitalization risk, mortality risk, and the expected clinical risk profile of patients presenting with symptoms suggestive of HF [16,17]. Underlying disease status was represented using epidemiologic inputs informed by population-based Chinese data and, where possible, calibrated to registry-based evidence relevant to populations with symptomatic HF.

Two diagnostic strategies were compared: (1) an exploratory age-specific NT-proBNP strategy using operational thresholds of 100 ng/L for patients aged <65 years and 248 ng/L for patients aged ≥65 years and (2) a conventional threshold strategy using a cutoff of 125 ng/L as the comparator [18]. Test results at or above the applicable cutoff were classified as positive. The thresholds of 100/248 ng/L were selected as exploratory, literature-informed thresholds based on prior Chinese studies of age-related NT-proBNP reference ranges in apparently healthy adults [10,11,19]. These values should not be interpreted as directly validated diagnostic cutoffs in symptomatic patients with suspected HF or as guideline-endorsed thresholds.

### Decision Tree

The decision tree represented only the initial diagnostic phase. Patients entered the tree and were classified as true positive (TP), false negative (FN), false positive (FP), or true negative (TN) according to their test results and underlying disease status, consistent with previous diagnostic-strategy modeling approaches in primary care HF evaluation [20].

TP patients were assumed to have HF correctly identified at the initial testing stage. FN patients were assumed to have HF that was not identified initially and, therefore, entered a delayed-diagnosis pathway. FP patients were assumed not to have HF but to undergo additional downstream assessment before HF was ruled out. TN patients were assumed not to have HF and entered the non-HF pathway directly.

In the base-case analysis, echocardiography plus specialist clinical assessment was modeled as the downstream reference diagnostic pathway, with substantially higher diagnostic accuracy than NT-proBNP screening. Residual misclassification at this stage was not explicitly modeled in the base case. This simplifying assumption was considered qualitatively in the Limitations section and further explored in scenario analyses.

### Markov Model Structure

After the diagnostic decision tree, patients entered long-term state-transition pathways representing HF-related and non-HF–related outcomes. The model used monthly cycles over a 10-year time horizon, with cycle-length and within-cycle correction considerations informed by standard pharmacoeconomic modeling practice [21]. Death was modeled as an absorbing state in all pathways.

The HF pathway was designed to reflect the fact that diagnosis does not necessarily imply immediate hospitalization. Accordingly, patients with recognized HF entered a diagnosed stable HF state rather than an HF hospitalization state by default. This pathway included diagnosed stable HF, HF hospitalization, posthospital HF, and death. Patients in the diagnosed stable HF state could remain stable, experience an HF hospitalization, or die. Patients who survived hospitalization entered a posthospital HF state characterized by intensified follow-up and elevated risks of rehospitalization and mortality. From this state, they could transition back to diagnosed stable HF, undergo rehospitalization, or die.

Initially, FN patients entered an undiagnosed HF state rather than being assumed to undergo deterministic re-presentation after a fixed interval. This state represented patients with underlying HF whose diagnosis had been delayed. Subsequent recognition could occur after a variable delay through repeat clinical presentation, worsening symptoms, hospitalization, or later clinical reassessment. Once recognized, these patients transitioned either to diagnosed stable HF if recognition occurred in the outpatient setting or to posthospital HF if HF was identified during hospitalization.

Patients without HF entered a weighted composite non-HF symptomatic pathway. This pathway was intended to represent adults undergoing evaluation for suspected HF who were ultimately found to have alternative non-HF causes of presentation. Because multiple alternative diagnoses may produce symptoms suggestive of HF, a pragmatic composite-state approach was used rather than separate disease-specific Markov models. The composite case mix was intended to reflect common alternative diagnoses encountered in routine practice, including chronic obstructive pulmonary disease, respiratory infection or pneumonia, obesity-related dyspnea, renal dysfunction, atrial fibrillation, myocardial ischemia, and related non-HF causes of breathlessness or edema [13,22]. The non-HF pathway included a non-HF symptomatic stable state, non-HF hospitalization, and death.

**Figure 1:**
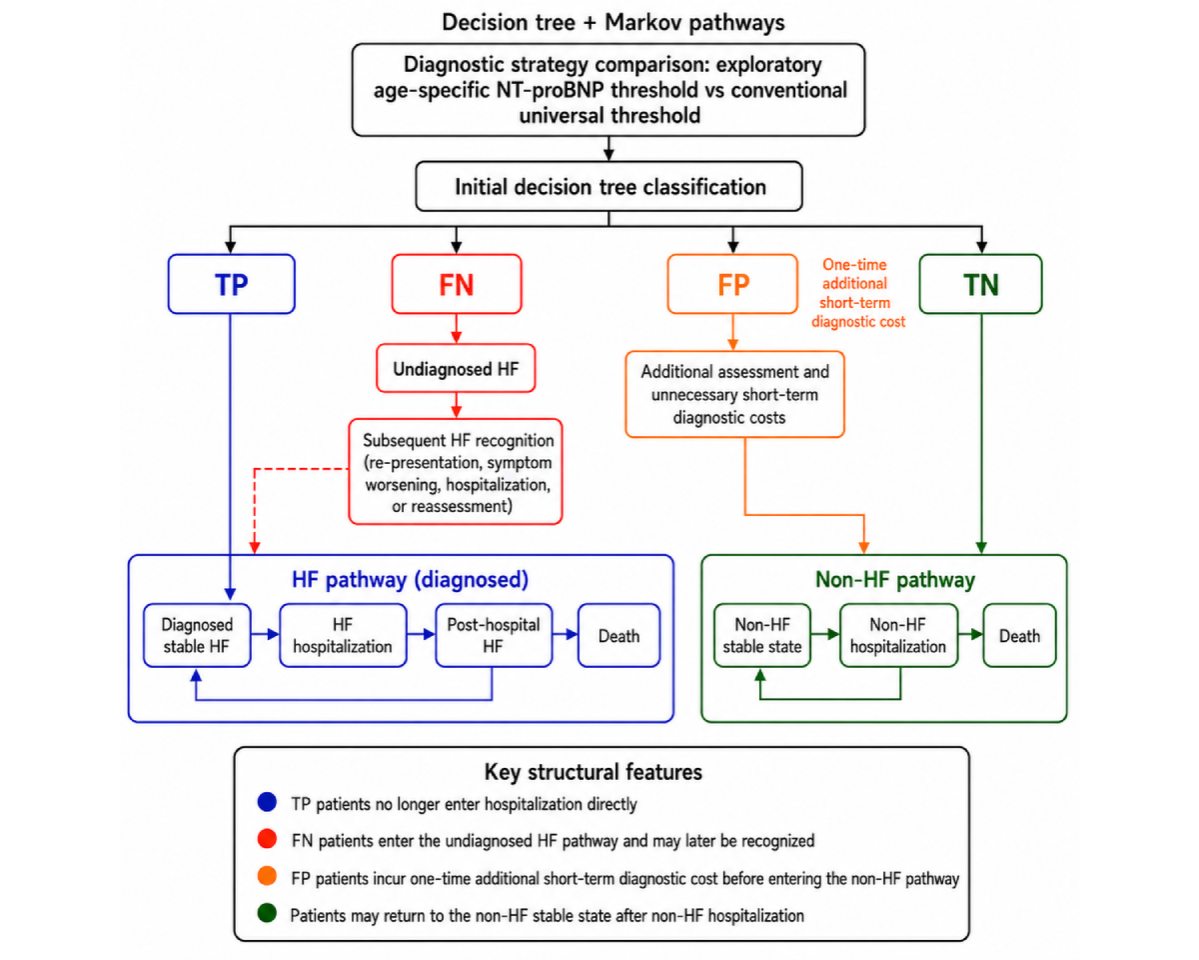
Final model structure of the diagnostic decision tree and Markov pathways. FN: false negative; FP: false positive; HF: heart failure; NT-proBNP: N-terminal pro-B-type natriuretic peptide; TN: true negative; TP: true positive.

FP and TN patients shared the same long-term non-HF pathway after the initial diagnostic phase. The difference between these groups was that FP patients incurred additional short-term costs associated with unnecessary consultation, echocardiography, and specialist assessment before HF was ruled out, whereas TN patients entered the non-HF pathway without these additional diagnostic costs. The final model structure is shown in Figure 1.

### Model Inputs

Diagnostic accuracy parameters for the conventional NT-proBNP strategy were informed by published diagnostic accuracy studies in patients with suspected HF [18,23]. Sensitivity and specificity were modeled using β distributions in the probabilistic sensitivity analysis (PSA). For the exploratory age-specific NT-proBNP strategy (100/248 ng/L), no directly matched diagnostic accuracy study was identified in a dedicated symptomatic Chinese cohort of patients with suspected HF. Accordingly, sensitivity and specificity for this strategy were specified as provisional literature-informed calibrated estimates rather than directly observed empirical values. The calibration reflected the expected directional change relative to the conventional threshold of 125 ng/L: the lower threshold in patients aged <65 years was expected to increase sensitivity and reduce specificity, whereas the higher threshold in patients aged ≥65 years was expected to reduce sensitivity and improve specificity. The resulting base-case estimates were set at 0.95 for sensitivity and 0.45 for specificity and were examined using wide uncertainty ranges, β distributions in PSA, and alternative diagnostic performance scenarios [10,11,19].

The selection of the provisional base-case estimates for the exploratory age-specific strategy was anchored to the reported diagnostic performance of the conventional 125 ng/L threshold (sensitivity 0.98 and specificity 0.35) and to the expected directional effects of lowering or increasing the threshold in the corresponding age groups. These estimates were not derived from a directly observed age-stratified diagnostic accuracy dataset. Rather, they were chosen a priori for the protocol-stage model to represent a conservative net diagnostic trade-off relative to the conventional strategy: a modest decrease in sensitivity from 0.98 to 0.95, together with a modest increase in specificity from 0.35 to 0.45. This specification reflects the hypothesis that the higher threshold in older adults may reduce FP classifications, while the overall sensitivity of the combined strategy may be slightly lower because some HF cases in older adults could be missed. Therefore, the base-case diagnostic accuracy values are intentionally treated as uncertain calibrated inputs rather than confirmed empirical parameters.

Transition probabilities for the HF pathway were derived, where available, from Chinese HF registries and published literature relevant to HF progression, hospitalization, and mortality [16,17]. Additional calibration was undertaken using external evidence from major HF studies when local data were incomplete. Parameters governing delayed recognition among initially FN patients were based on clinically plausible assumptions informed by previous diagnostic modeling studies and were further explored in scenario analyses.

For the non-HF pathway, hospitalization and mortality risks were represented using a composite symptomatic non-HF case mix based on published Chinese data where available. Hospitalization risk, mortality risk, costs, and utility values for this pathway were assigned using aggregated or weighted estimates for the composite symptomatic population with no HF, with Chinese evidence used whenever possible. When directly applicable Chinese data were unavailable, parameter values were informed by published literature, expert consultation, or transparent model-based assumptions. Alternative assumptions regarding the composition of the non-HF pathway were examined in scenario and sensitivity analyses.

Because this manuscript presents a protocol embedded within an ongoing project on the establishment and clinical evaluation of Chinese reference intervals for major chronic disease–related laboratory tests, some model inputs are not yet supported by fully mature project-specific empirical data. Where directly applicable Chinese evidence was unavailable at the protocol stage, parameters were specified using published literature, provisional literature-informed calibrated estimates, or transparent assumption-based values. These inputs will be updated in the final analysis as additional project-specific Chinese data become available.

Detailed model inputs, including diagnostic accuracy parameters, transition probabilities, cost parameters, health utility parameters, and global model settings, are summarized in Multimedia Appendix 2.

### Costs and Health Outcomes

The economic evaluation was conducted from the perspective of the Chinese health care system and therefore included only direct medical costs related to diagnostic testing, specialist assessment, follow-up, disease management, and hospitalization. Diagnostic costs included NT-proBNP testing, echocardiography, and specialist consultation, where applicable. Patients in the diagnosed stable HF state incurred ongoing HF management costs, patients experiencing HF hospitalization incurred acute hospitalization costs, and patients in the posthospital HF state incurred follow-up costs after discharge. Patients in the non-HF pathway incurred non-HF management or hospitalization costs, as appropriate.

FP patients were not assumed to receive routine long-term HF-specific pharmacotherapy in the base-case analysis. Rather, they incurred additional short-term diagnostic and assessment costs associated with unnecessary downstream evaluation before entering the non-HF pathway. For FN patients, the monthly cost assigned to the undiagnosed HF state was not intended to represent higher formal HF management costs; rather, it reflected limited or nonstandard care before HF recognition. The economic consequences of delayed diagnosis were captured primarily through the higher risks of HF hospitalization and death in the undiagnosed HF state, rather than through a fixed one-time exacerbation penalty or an increased routine monthly state cost. Once hospitalized, these patients incurred acute HF hospitalization costs and then entered the posthospital HF state, where they accrued follow-up costs and remained at elevated risks of rehospitalization and mortality.

Costs related to implementation of the diagnostic strategy, including training, workflow adaptation, and administrative infrastructure, as well as broader societal costs such as productivity loss, informal care, transportation, and patient time, were not included in the base-case analysis [24].

Health outcomes were measured in quality-adjusted life-years (QALYs). Utility values were assigned to each health state, including diagnosed stable HF, HF hospitalization, posthospital HF, undiagnosed HF, non-HF symptomatic stable, and non-HF hospitalization. The disutility associated with hospitalization was captured through lower utility weights during the relevant cycle. Utility inputs were informed, where available, by published HF utility estimates in Asian populations and Chinese population-based utility norms [25-27].

### Discounting, Time Horizon, and Willingness-to-Pay Threshold

All costs were expressed in 2023 Chinese Yuan. The willingness-to-pay threshold was based on the 2023 gross domestic product per capita to ensure consistency with the selected cost year. Costs and health outcomes were discounted at an annual rate of 5% in accordance with the 2020 China Guidelines for Pharmacoeconomic Evaluations, and alternative discount rates were examined in sensitivity analyses [25,28,29].

The base-case analysis used a 10-year time horizon to capture the medium- to long-term consequences of diagnostic classification, including delayed recognition, hospitalization, posthospital progression, and mortality, while limiting additional uncertainty associated with extrapolation beyond the period supported by directly relevant evidence. A lifetime-horizon scenario analysis was planned to assess whether the cost-effectiveness conclusions were sensitive to the choice of analytic time horizon.

### Uncertainty and Sensitivity Analyses

Deterministic one-way sensitivity analyses will vary individual parameters across prespecified plausible ranges while holding all other parameters constant. PSA will be conducted using 5000 Monte Carlo simulations. In each simulation, uncertain model parameters will be sampled simultaneously from their assigned probability distributions, and mean costs, QALYs, and incremental cost-effectiveness ratios (ICERs) will be estimated [30,31].

In PSA, β distributions will be assigned to probabilities and utility values, γ distributions will be assigned to cost parameters, and log-normal distributions will be assigned to relative effect measures, where applicable. Scenario analyses will be used to explore structural uncertainty, including alternative assumptions regarding delayed recognition among initially FN patients, alternative NT-proBNP thresholds and their associated sensitivity and specificity estimates, alternative age distributions, downstream diagnostic management after a positive NT-proBNP result, and the composition of the non-HF pathway [32].

Because uncertainty around the estimated diagnostic accuracy of the exploratory age-specific strategy may materially influence model results, prespecified diagnostic performance scenarios will be conducted in addition to deterministic and probabilistic sensitivity analyses. These scenarios are intended to assess whether the incremental costs, QALYs, ICERs, and cost-effectiveness acceptability results are robust to alternative plausible assumptions regarding sensitivity and specificity. The diagnostic performance scenarios will include a base-case scenario (sensitivity 0.95 and specificity 0.45), a conservative scenario (sensitivity 0.90 and specificity 0.35), a neutral or no-specificity-gain scenario (sensitivity 0.95 and specificity 0.35), and an optimistic scenario (sensitivity 0.98 and specificity 0.60). All diagnostic performance scenarios will be analyzed using the same model structure and cost, utility, and transition-probability inputs as the base-case analysis, so that differences in results can be attributed to the assumed diagnostic performance of the age-specific strategy. The conclusions will be interpreted as robust only if the age-specific strategy remains cost-effective across clinically plausible diagnostic performance scenarios; otherwise, the conclusions will be presented as conditional on future empirical diagnostic validation.

To address uncertainty related to provisional and assumption-based parameters, all such inputs will be examined in deterministic one-way sensitivity analyses, PSA, and scenario analyses. Key parameters will be updated in the final analysis when project-specific Chinese data become fully available. In addition, prespecified age-stratified subgroup analyses will be conducted to assess whether cost-effectiveness results differ across clinically relevant age groups aligned with the diagnostic strategy.

### Model Validation

Model validation will include face validity assessment, internal calibration, and external validation. Face validity of the conceptual model, structural assumptions, and analytic plan was reviewed by experts in cardiology and health economics or health services management.

For calibration, prespecified calibration targets will include the prevalence of underlying HF in the symptomatic suspected-HF cohort, HF hospitalization rates, posthospital rehospitalization rates, all-cause mortality in diagnosed HF, mortality after HF hospitalization, and the distribution of diagnostic classifications, where empirical data are available. For the non-HF pathway, calibration will focus on the overall hospitalization and mortality risks of the composite symptomatic population with no HF rather than on disease-specific calibration for each alternative diagnosis. Model-predicted outputs will be compared with Chinese registry data, published Chinese studies, and project-specific data, when available.

As an a priori criterion, calibration will be considered acceptable if predicted proportions differ from target values by no more than 5 absolute percentage points or if predicted event rates and costs fall within 20% of the target estimate or within the reported CI or plausible range, when available. These criteria are intended to provide transparent boundaries for assessing model plausibility while recognizing that the target population and data sources may not be perfectly identical.

If calibration targets are not met, only prespecified parameters with clinical and empirical uncertainty will be adjusted within their predefined ranges. These may include underlying HF prevalence, transition probabilities for HF hospitalization and death, delayed recognition probabilities among initially FN patients, posthospital rehospitalization and mortality probabilities, and composite non-HF hospitalization and mortality risks. Diagnostic threshold definitions, cycle length, analytic perspective, discount rate, and willingness-to-pay thresholds will not be adjusted during calibration. All calibration adjustments and their rationale will be documented transparently in the final analysis.

For external validation, model-predicted outcomes will be compared with independent published studies, Chinese registry-based data, and project-specific datasets that were not used for initial parameterization, whenever available. External validation will focus on whether the model reproduces clinically plausible patterns of HF hospitalization, posthospital outcomes, mortality, non-HF hospitalization, and overall costs in comparable Chinese populations.

Because the model draws on multiple data sources and includes several assumption-based parameters, validation is intended to assess the overall plausibility and consistency of predicted outcomes rather than exact concordance with any single study population.

### Ethical Considerations

This study protocol was approved by the Medical Ethics Committee of China Medical University on October 16, 2024 (approval number: [2021] 2024-920-2). The model is based on published and secondary data sources and does not involve direct participant intervention.

## Results

This is a study protocol. Funding has been obtained from the Noncommunicable Chronic Diseases-National Science and Technology Major Project. Model construction has been completed, and parameter collection, calibration, and validation are ongoing. The final cost-effectiveness analysis is expected to be completed after project-specific Chinese data become available. Planned outputs include total costs, QALYs, ICERs, diagnostic classification outcomes under each strategy, and cost-effectiveness acceptability results.

## Discussion

### Anticipated Findings

This protocol describes an economic evaluation of diagnostic strategies rather than a treatment-comparison model for established HF. The model structure was designed to separate initial diagnostic classification from downstream disease progression, thereby avoiding the overstatement of the clinical severity or cost consequences of a positive NT-proBNP result alone. By linking an initial decision tree to long-term state-transition pathways, the model is intended to capture how correct diagnosis, delayed diagnosis, unnecessary downstream assessment, hospitalization, and death may translate into differences in costs and QALYs across alternative diagnostic threshold strategies.

This protocol addresses a clinically and policy-relevant question in China. NT-proBNP is widely used in the evaluation of HF, yet commonly applied thresholds have largely been derived from manufacturer-based or non-Chinese evidence. Previous Chinese reference-range studies suggest age-related variation in NT-proBNP distributions, providing a rationale for evaluating whether a single universal threshold is appropriate for Chinese adults undergoing evaluation for HF. Accordingly, this protocol is designed to assess an exploratory age-specific threshold strategy while explicitly recognizing that these thresholds have not been formally adopted in national guidelines and remain subject to uncertainty.

A major strength of the protocol is the explicit linkage between diagnostic classification and downstream clinical and economic consequences. Rather than treating test performance as an isolated end point, the model connects TP, FN, FP, and TN results to subsequent HF-related or non-HF–related pathways. This framework allows the evaluation of not only immediate diagnostic outcomes but also later hospitalization, delayed recognition, follow-up management, and health-related quality of life. The protocol also incorporates multiple approaches to uncertainty analysis, including deterministic sensitivity analysis, PSA, and structural scenario analyses.

Some model inputs remain provisional at the protocol stage. Where directly applicable Chinese evidence is unavailable, parameters have been specified using published literature, literature-informed calibrated estimates, or transparent assumption-based values. These parameters will be updated in the final analysis as project-specific Chinese data become available.

### Limitations

The age-specific NT-proBNP thresholds evaluated in this study were informed by previous Chinese age-related reference-range research in apparently healthy adults and should therefore be interpreted as exploratory, literature-informed operational thresholds rather than formally guideline-endorsed diagnostic cutoffs. Their external validity in symptomatic adults with suspected HF remains uncertain and will require further prospective validation in dedicated Chinese diagnostic cohorts.

The model does not explicitly represent severity-based HF states such as New York Heart Association classes. Rather, it captures average downstream progression through diagnosed stable HF, HF hospitalization, posthospital HF, undiagnosed HF, and death. This parsimonious structure was selected because the primary objective was to evaluate diagnostic strategies in patients with suspected HF rather than treatment strategies in established HF, consistent with decision-analytic modeling principles and previous threshold-based HF diagnostic economic models. However, this simplified approach does not fully capture within-HF severity heterogeneity.

Several model inputs remain provisional at the current stage. Although these parameters were specified transparently and examined through extensive uncertainty analyses, the final cost-effectiveness results will depend on subsequent updating of key diagnostic, epidemiologic, and cost inputs as project-specific Chinese data mature.

The base-case model did not explicitly model residual misclassification after echocardiography plus specialist clinical assessment. Although this downstream pathway was assumed to have substantially higher diagnostic accuracy than NT-proBNP screening, it is not perfectly accurate in routine clinical practice. This simplifying assumption may underestimate the consequences of residual diagnostic uncertainty after downstream assessment. Therefore, the impact of imperfect downstream diagnostic accuracy will be examined in scenario analyses.

The FN pathway was modeled using an undiagnosed HF state with variable subsequent recognition rather than a fixed empirical schedule of re-presentation. This approach was intended to avoid an overly deterministic representation of delayed diagnosis, but it also means that some downstream transition parameters rely on clinically plausible assumptions rather than directly observed evidence.

The non-HF pathway was modeled as a weighted composite symptomatic pathway rather than as separate disease-specific pathways. Accordingly, the estimated costs, utilities, hospitalization risks, and mortality risks in this branch should be interpreted as average values for a representative symptomatic comparator population with no HF rather than as disease-specific estimates.

The base-case analysis adopted a health care system perspective and did not include implementation costs or broader societal costs. Consequently, the model may not fully capture the wider economic implications of introducing a new diagnostic strategy, particularly with respect to productivity loss, informal care, transportation, and patient time.

The base-case model used a 10-year time horizon rather than a lifetime horizon. Although this choice was intended to reduce uncertainty associated with long-term extrapolation, it may not fully capture all downstream benefits or harms over the remaining lifetime of patients. Lifetime-horizon scenario analyses are therefore planned.

### Conclusions

By linking diagnostic classification to downstream clinical progression and health care costs, this protocol provides a transparent framework for evaluating whether exploratory age-specific NT-proBNP thresholds may offer clinical or economic advantages over a conventional universal threshold strategy in China. The final analysis is intended to inform future research and policy while explicitly acknowledging the uncertainty surrounding exploratory threshold selection, provisional model inputs, and other structural assumptions.

## Appendix

Multimedia Appendix 1 CHEERS (Consolidated Health Economic Evaluation Reporting Standards) 2022 checklist for economic evaluation.

Multimedia Appendix 2 Model input parameters.

## Abbreviations

CHEERS: Consolidated Health Economic Evaluation Reporting Standards
FN: false negative
FP: false positive
HF: heart failure
ICER: incremental cost-effectiveness ratio
NT-proBNP: N-terminal pro-B-type natriuretic peptide
PSA: probabilistic sensitivity analysis
QALY: quality-adjusted life-year
TN: true negative
TP: true positive
