# Supplementary material for: Cost-Effectiveness Analysis of Age-Specific N-Terminal Pro-B-Type Natriuretic Peptide Thresholds for Heart Failure Diagnosis in China: Protocol for a Markov Model–Based Study
Source: JMIR Res Protoc. 2026 Jun 29;15:e95071. doi: 10.2196/95071 (PMC13365891; doi:10.2196/95071)
Supplement: Multimedia Appendix 1 [file resprot_v15i1e95071_app1.docx]

**CHEERS 2022 Checklist**

| **Item** | **No.** | **Guidance for reporting** | **Reported in section** |
| --- | --- | --- | --- |
| **TITLE** | | | |
| Title | 1 | Identify the study as an economic evaluation and specify the interventions being compared. | Title |
| **ABSTRACT** | | | |
| Abstract | 2 | Provide a structured summary that highlights context, key methods, results and alternative analyses. | Abstract |
| **INTRODUCTION** | | | |
| Background and objectives | 3 | Give the context for the study, the study question and its practical relevance for decision making in policy or practice. | Introduction |
| **METHODS** | | | |
| Health economic analysis plan | 4 | Indicate whether a health economic analysis plan was developed and where available. | Methods: Model Overview; Model Inputs; Uncertainty and Sensitivity Analyses; Model Validation |
| Study population | 5 | Describe characteristics of the study population, such as age range, demographics, socioeconomic, or clinical characteristics. | Methods: Target Population and Diagnostic Strategies |
| Setting and location | 6 | Provide relevant contextual information that may influence findings. | Methods: Target Population and Diagnostic Strategies; Costs and Health Outcomes |
| Comparators | 7 | Describe the interventions or strategies being compared and why chosen. | Methods: Target Population and Diagnostic Strategies |
| Perspective | 8 | State the perspective(s) adopted by the study and why chosen. | Methods: Costs and Health Outcomes |
| Time horizon | 9 | State the time horizon for the study and why appropriate. | Methods: Discounting, Time Horizon, and Willingness-to-Pay Threshold |
| Discount rate | 10 | Report the discount rate(s) and reason chosen. | Methods: Discounting, Time Horizon, and Willingness-to-Pay Threshold; Multimedia Appendix 2 |
| Selection of outcomes | 11 | Describe what outcomes were used as the measure(s) of benefit(s) and harm(s). | Abstract; Methods: Costs and Health Outcomes; Results |
| Measurement of outcomes | 12 | Describe how outcomes used to capture benefit(s) and harm(s) were measured. | Methods: Decision Tree; Markov Model Structure; Model Inputs |
| Valuation of outcomes | 13 | Describe the population and methods used to measure and value outcomes. | Methods: Costs and Health Outcomes; Multimedia Appendix 2 |
| Measurement and valuation of resources and costs | 14 | Describe how costs were valued. | Methods: Costs and Health Outcomes; Multimedia Appendix 2 |
| Currency, price date, and conversion | 15 | Report the dates of the estimated resource quantities and unit costs, plus the currency and year of conversion. | Methods: Discounting, Time Horizon, and Willingness-to-Pay Threshold; Multimedia Appendix 2 |
| Rationale and description of model | 16 | If modelling is used, describe in detail and why used. Report if the model is publicly available and where it can be accessed. | Methods: Model Overview; Markov Model Structure; Figure 1 |
| Analytics and assumptions | 17 | Describe any methods for analysing or statistically transforming data, any extrapolation methods, and approaches for validating any model used. | Methods: Decision Tree; Markov Model Structure; Model Inputs; Model Validation |
| Characterizing heterogeneity | 18 | Describe any methods used for estimating how the results of the study vary for sub-groups. | Methods: Uncertainty and Sensitivity Analyses |
| Characterizing distributional effects | 19 | Describe how impacts are distributed across different individuals or adjustments made to reflect priority populations. | Not applicable; distributional/equity analysis was not planned in this protocol |
| Characterizing uncertainty | 20 | Describe methods to characterize any sources of uncertainty in the analysis. | Methods: Uncertainty and Sensitivity Analyses; Limitations |
| Approach to engagement with patients and others affected by the study | 21 | Describe any approaches to engage patients or service recipients, the general public, communities, or stakeholders (eg, clinicians or payers) in the design of the study. | Methods: Model Validation |
| **RESULTS** | | | |
| Study parameters | 22 | Report all analytic inputs, such as values, ranges, references, and uncertainty or distributional assumptions. | Multimedia Appendix 2 |
| Summary of main results | 23 | Report the mean values for the main categories of costs and outcomes of interest and summarize them in the most appropriate overall measure. | Results (planned outputs; study protocol) |
| Effect of uncertainty | 24 | Describe how uncertainty about analytic judgments, inputs, or projections affect findings. Report the effect of choice of discount rate and time horizon, if applicable. | Methods: Uncertainty and Sensitivity Analyses; Results (planned outputs); Limitations |
| Effect of engagement with patients and others affected by the study | 25 | Report on any difference patient/service recipient, general public, community, or stakeholder involvement made to the approach or findings of the study. | Methods: Model Validation |
| **DISCUSSION** | | | |
| Study findings, limitations, generalizability, and current knowledge | 26 | Report key findings, limitations, ethical or equity considerations not captured, and how these could impact patients, policy, or practice. | Discussion; Limitations; Conclusions |
| **OTHER RELEVANT INFORMATION** | | | |
| Source of funding | 27 | Describe how the study was funded and any role of the funder in the identification, design, conduct, and reporting of the analysis. | Funding |
| Conflicts of interest | 28 | Report authors conflicts of interest according to journal or International Committee of Medical Journal Editors requirements. | Conflict-of-interest disclosure in JMIR submission forms; no conflicts reported in the manuscript file |

**Reference:** Husereau D, Drummond M, Augustovski F, de Bekker-Grob E, Briggs AH, Carswell C, Caulley L, Chaiyakunapruk N, Greenberg D, Loder E, Mauskopf J, Mullins CD, Petrou S, Pwu RF, Staniszewska S; CHEERS 2022 ISPOR Good Research Practices Task Force. Consolidated Health Economic Evaluation Reporting Standards 2022 (CHEERS 2022) Statement: Updated Reporting Guidance for Health Economic Evaluations. BMJ. 2022;376:e067975. The checklist is Open Access under the Creative Commons Attribution (CC BY 4.0) license: http://creativecommons.org/licenses/by/4.0/.
