# Supplementary material for: Cost-Effectiveness Analysis of Age-Specific N-Terminal Pro-B-Type Natriuretic Peptide Thresholds for Heart Failure Diagnosis in China: Protocol for a Markov Model–Based Study
Source: JMIR Res Protoc. 2026 Jun 29;15:e95071. doi: 10.2196/95071 (PMC13365891; doi:10.2196/95071)
Supplement: Multimedia Appendix 2 [file resprot_v15i1e95071_app2.docx]

**Model input parameters.**

| **Parameters** | **Base-case value** | **Range or uncertainty** | **Distribution** | **Source or rationale** |
| --- | --- | --- | --- | --- |
| **Diagnostic accuracy parameters** | | | | |
| Sensitivity of conventional NT-proBNP^a^ threshold (125 ng/L) | 0.98 | Reported study value; plausible range in sensitivity analyses | Beta | [18] |
| Specificity of conventional NT-proBNP threshold (125 ng/L) | 0.35 | Reported study value; plausible range in sensitivity analyses | Beta | [18] |
| Sensitivity of exploratory age-specific NT-proBNP strategy (100/248 ng/L)^f^ | 0.95 | 0.90-0.98 | Beta | Provisional literature-informed calibrated estimate based on prior Chinese age-related NT-proBNP reference-range research [10,11,19] |
| Specificity of exploratory age-specific NT-proBNP strategy (100/248 ng/L)^f^ | 0.45 | 0.35-0.60 | Beta | Provisional literature-informed calibrated estimate based on prior Chinese age-related NT-proBNP reference-range research [10,11,19] |
| Prevalence of underlying HF^b,g^ in the symptomatic cohort | 0.32 | 0.20-0.45 | Beta | Literature-informed symptomatic-cohort estimate based on suspected-HF diagnostic studies [18,20,22] |
| **Transition probabilities** | | | | |
| Monthly probability of HF hospitalization from diagnosed stable HF | 0.015 | 0.010-0.025 | Beta | Literature-informed estimate anchored to Chinese HF registry evidence [16,17] |
| Monthly probability of death from diagnosed stable HF | 0.005 | 0.003-0.010 | Beta | Literature-informed estimate anchored to Chinese HF registry evidence [16,17] |
| Monthly probability of return to diagnosed stable HF from posthospital HF | 0.25 | 0.15-0.40 | Beta | Assumption based |
| Monthly probability of rehospitalization from posthospital HF | 0.08 | 0.05-0.15 | Beta | Literature-informed estimate anchored to Chinese HF registry evidence [16,17] |
| Monthly probability of death from posthospital HF | 0.02 | 0.01-0.04 | Beta | Literature-informed estimate anchored to Chinese HF registry evidence [16,17] |
| Monthly probability of HF hospitalization from undiagnosed HF | 0.02 | 0.012-0.035 | Beta | Assumption based |
| Monthly probability of death from undiagnosed HF | 0.006 | 0.003-0.012 | Beta | Assumption based |
| Monthly probability of subsequent recognition among initially false-negative patients | 0.08 | 0.03-0.15 | Beta | Assumption based |
| Monthly probability of non-HF hospitalization from non-HF stable state | 0.01 | 0.005-0.020 | Beta | Composite non-HF comparator estimate informed by suspected-HF differential-diagnosis literature [13,22] |
| Monthly probability of death from non-HF stable state | 0.002 | 0.001-0.005 | Beta | Composite non-HF comparator estimate informed by suspected-HF differential-diagnosis literature [13,22] |
| Monthly probability of return to non-HF stable state after non-HF hospitalization | 0.30 | 0.20-0.45 | Beta | Assumption based |
| Monthly probability of death from non-HF hospitalization | 0.01 | 0.005-0.025 | Beta | Assumption based |
| **Cost parameters** | | | | |
| NT-proBNP test cost | 150 | 100-250 | Gamma | Local fee schedule or hospital pricing^c^ |
| Echocardiography cost | 250 | 180-400 | Gamma | Local fee schedule or hospital pricing^c^ |
| Specialist consultation cost | 100 | 50-200 | Gamma | Local fee schedule or hospital pricing^c^ |
| Monthly cost of diagnosed stable HF | 500 | 300-800 | Gamma | Chinese HF registry and literature-informed costing [16,17] |
| Cost per HF hospitalization | 28000 | 20000-40000 | Gamma | Chinese HF registry and literature-informed costing [16,17] |
| Monthly cost of posthospital HF follow-up | 700 | 400-1000 | Gamma | Assumption based |
| Monthly cost of undiagnosed HF | 400 | 200-700 | Gamma | Assumption based |
| Monthly cost of non-HF stable state | 300 | 150-600 | Gamma | Composite non-HF comparator estimate |
| Cost per non-HF hospitalization | 10000 | 6000-18000 | Gamma | Composite non-HF comparator estimate |
| Additional short-term diagnostic cost for false-positive patients | 350 | 200-700 | Gamma | Downstream reassessment cost estimate |
| **Health utility parameters** | | | | |
| Utility of diagnosed stable HF | 0.871 | 0.80-0.90 | Beta | [26] |
| Utility of HF hospitalization | 0.215 | 0.15-0.35 | Beta | [26] |
| Utility of posthospital HF | 0.800 | 0.75-0.87 | Beta | Assumption based |
| Utility of undiagnosed HF | 0.820 | 0.75-0.87 | Beta | Assumption based |
| Utility of non-HF stable state^h^ | 0.850 | 0.80-0.95 | Beta | Chinese population utility norms, conservatively adjusted for symptomatic evaluation population [27] |
| Utility of non-HF hospitalization | 0.600 | 0.50-0.75 | Beta | Assumption based |
| Utility of death | 0 | — | — | Absorbing state |
| **Global model settings** | | | | |
| Cycle length | 1 month | — | — | Model design |
| Time horizon | 10 years | Lifetime scenario analysis | — | Base case plus scenario analysis |
| Annual discount rate for costs | 5% | 0%-8% in sensitivity analyses | — | [28] |
| Annual discount rate for QALYs^d^ | 5% | 0%-8% in sensitivity analyses | — | [28] |
| Willingness-to-pay threshold | CNY 89,358 (US $13,199)^e^ per QALY | Alternative thresholds in scenario analyses | — | [25] |
| Number of probabilistic sensitivity analysis simulations | 5000 | — | — | Prespecified; informed by convergence guidance [30] |

^a^ NT-proBNP: N-terminal pro-B-type natriuretic peptide.

^b^ HF: heart failure.

^c^ Diagnostic test and consultation costs are protocol-stage base-case estimates and plausible ranges derived from local fee schedules and hospital price disclosures. In the formal analysis, parameter values will be updated, where available, using the most applicable official price standards for the target setting, such as provincial or municipal medical service price schedules, medical insurance price catalogues, or hospital prices implementing local guidance.

^d^ QALY: quality-adjusted life-year.

^e^ All costs are presented in Chinese Yuan (CNY) unless otherwise indicated. Currency conversion was based on CNY 1=US $0.1477 as of June 19, 2026.

^f^ The operational thresholds of 100/248 ng/L were selected as exploratory age-specific thresholds informed by prior Chinese research on age-related NT-proBNP reference ranges in apparently healthy adults. These thresholds have not been directly validated in a dedicated cohort of symptomatic Chinese patients with suspected HF. Accordingly, sensitivity and specificity were modeled as provisional literature-informed calibrated estimates. The calibration reflected the expected direction of change relative to the conventional 125 ng/L threshold: the lower threshold in patients aged <65 years was expected to increase sensitivity and reduce specificity, whereas the higher threshold in patients aged ≥65 years was expected to reduce sensitivity and improve specificity. These estimates were examined in deterministic, probabilistic, and scenario analyses.

^g^ The prevalence of underlying HF in the symptomatic cohort was specified as a literature-informed base-case estimate for populations with suspected HF and will be calibrated against Chinese registry and published outcome data, where available.

^h^ The utility of the non-HF stable state was informed by Chinese population-based health utility norms and conservatively adjusted to reflect symptomatic individuals undergoing evaluation for suspected HF.

Em dashes indicate not applicable.
